# Community Knowledge, Perceived Beliefs and Associated Factors of Mental Distress: A Case Study from Northern Ethiopia

**DOI:** 10.3390/ijerph15112423

**Published:** 2018-10-31

**Authors:** Aradom Gebrekidan Abbay, Alemayehu Tibebe Mulatu, Hossein Azadi

**Affiliations:** 1 Department of Cooperative Studies, Mekelle University, 231 Mekelle, Ethiopia; 2 Tigrai Regional Health Bureau, Seqota Declaration, Regional Program Delivery Unit, 7 Mekelle, Ethiopia; acommet@gmail.com; 3 Research Group Climate Change and Security, Institute of Geography, University of Hamburg, 21073 Hamburg, Germany; 4 Department of Geography, Ghent University, 9000 Ghent, Belgium; hossein.azadi@ugent.be

**Keywords:** community health, mental distress, perceived beliefs, community knowledge, MAKS, community prevalence

## Abstract

*Background*: All of society is affected by mental health problems, not just a minor, isolated part. Mental health problems represent a major challenge to the global development of community health. This study examined the community health knowledge, perceived beliefs, and associated factors of mental distress (MD) in Mekelle city in Northern Ethiopia. *Methods*: The current study used a cross-sectional approach calculating a sample of 260 adults living in the two sub-cities of Mekelle city. To select the sample households in each sub-city, systematic random sampling was used. Self-reported questionnaire (SRQ-20 with a cutoff point of 7), and Mental Health Knowledge Schedule (MAKS) instruments were included within the structured questionnaire tool to clarify community occurrence and the level of health mental knowledge. *Results*: The likelihood of having MD was higher among the study participants who were female, employed, self-employed, and daily alcohol and khat users. The results also showed that the level of mental health knowledge among the participants was low. *Conclusions*: Factors such as being male, having a higher level of education, and having strong levels of social support were found to be the independent predictors of good mental health and community mental health knowledge.

## 1. Introduction

All of society is affected by mental health problems, not just a small, isolated part. They are a serious challenge to global community health and development. There are no groups of human beings that are immune to developing mental distress (MD). The risk of suffering from MD is higher among the poor, homeless, the unemployed, those with little education, victims of violence, migrants and displaced people, native populations, children and young people, abused women, and the abandoned elderly [1].

There is chance of developing mental or neurological disorders for one in four people in the world at some point during their lives. Currently, about 450 million people are dealing with these issues, with MD among the leading causes of morbidity and disability worldwide. About 12% of the global burden of disease is linked with mental and behavioral disorders. Only 1% of total budget is allocated to the mental health budget or expenditure in the majority of countries (both developed and developing). There is a clear disproportionate relationship between disease burden and disease spending [2]. According to WHO’s Global Burden of Disease [3], neuropsychiatric disorders constitute 33% of the years living with disability (YLD).

About a third of global Disability Adjusted Life Years (DALYs) were found in China and India (66 million DALY) [4] which is attributable to mental, neurological, and substance use distress. This figure is larger than that in all developed countries combined (50 million DALYs).

In sub-Saharan Africa including Ethiopia, rates of psychological distress in adults are particularly elevated. As it is clearly stated in the national mental health strategy of Ethiopia (2012) [5], mental disease is the leading key to non-communicable disorder in terms of burden in Ethiopia. About 11% of the total burden of disease is affected a predominantly rural area of Ethiopia. In the top ten most burdensome conditions, schizophrenia and depression are listed, out-ranking HIV/AIDS. These shocking statistics demonstrate that mental illnesses have been neglected as a main health priority in Ethiopia.

However, there is limited information about the perception and the attitude of the public regarding mental health problems [6]. A study conducted in Borana, Ethiopia revealed drivers with respect to supernatural causes such as perceptions of being controlled by evil spirits, being cursed, bewitched, having ‘exposure to wind’, and subsequent attacks by evil spirits in postnatal women. Moreover, bio-psychosocial causes such as infections (malaria), loss, ‘over-thinking’, and alcohol and khat abuse are the leading causes of mental disturbance in Ethiopia [7,8].

Other studies also showed that regarding mental illness the main factor leading to stigmatization and labeling is linked with people’s belief. A significant barrier to positive outcomes across cultures and nations is stigma against people with mental illness. This is relevant to the level of threat of mental symptoms, intolerance towards diversity, and inaccurate perceptions of MD [9,10].

However, there are only a limited number of studies conducted in Ethiopia and specifically in the Tigrai region context examining community health knowledge and beliefs about MD or mental illness. Therefore, this study examined community knowledge, perceived beliefs and associated factors of mental illness in Mekelle city, Tigrai, Ethiopia. The study also investigated the prevalence and associated risk factors of MD to fill a huge perceived research gap in this area.

## 2. Definition of Mental Health and the Major Mental Illness: An Overview

Mental health is considered as the vital asset to human health and wellbeing. However, this term is narrowly defined, and may be conceptualized as the absence of mental illness. The terms “mental health problems” and “mental illness” link with the range of cognitive, emotional and behavioral disorders. These issues interfere with the lives and productivity of people. They are the most commonly reported type of mental health complaint, and are commonly the result of a reaction to life stresses or negative life experiences [11].

According to the Hunter Institute of Mental health (2013) [12] “mental health is a positive concept relevant to the social and emotional wellbeing of people and societies.” As Bland et al. (2009) [13] pointed out, mental illness does not occur in biological or genetic isolation; it takes place due to the messy reality of our lives (epidemiological factors).

Today, mental health problems are recognized as a public health problem in developed and developing countries [6]. How people think and how they perceive their environments are altered by mental problems and illness. This can affect levels of hope, trust, self-efficacy, and personal relationships, and results in significant impacts on community wellbeing. Mental illness puts at risk the construction of our identities at a time when we are trying to find our personalities in relation to ourselves, others, and the wider society [13,14].

Low level of mental health literacy is the one of the causes of high incidence [15]. Based on conducted study by Jorm (2000) [16] the term ‘mental health literacy’ has been defined as “knowledge and beliefs about MD which help their recognition, management or prevention”. Additionally, several components are linked with mental community health literacy, including: (a) being capable of recognizing specific disorders or different types of psychological distress; (b) recognizing risk factors and causes; (c) beliefs about self-help interventions; (d) knowledge and beliefs about professional help available; (e) attitudes which facilitate recognition and appropriate help-seeking; and (f) knowledge of how to follow mental health information.

Careful evaluation of the norms, beliefs and tradition within the individual’s cultural environment also belong to the recognition of MD [17]. In Ethiopia where poverty, war, famine, displacement, and homelessness are common, mental health is also becoming a major public health problem [18].

The following work will review studies on community health knowledge, perceived beliefs, and associated factors of mental illness. The review will take a deep dive on community knowledge and ideas about causes, symptoms of MD, public attitude and perception towards people with mental diseases. The review also provides empirical evidence on the prevalence of common MD and its associated factors. Therefore, this review has two major parts; the first part discusses the findings of different studies on community knowledge and perceived beliefs about MD. The second part of the review highlights and discusses evidence on the prevalence as well as associated factors of mental illness.

### 2.1. Mental Community Health Literacy

The high lifetime prevalence of MD (up to 50%) is drawing the attention of the public towards the importance of greater mental health literacy. The dimensions of mental health literacy in Western countries are totally different from some developing countries where more than half of the population may be illiterate. Supernatural causes of MD are more widely known. Traditional sources of help, (i.e., spiritual healers) are better accepted than medical advice in these countries [19]. This part of the review therefore emphasizes on community recognition of MD, beliefs about the etiology of mental illness, and help seeking, and community reaction to the mentally ill.

### 2.2. Recognition of Mental Disorders

Based on the conducted study in United Kingdom assessing community health knowledge of mental illness and reactions towards mentally ill people (215 participants) reported that 21% of respondents had no idea about different types of mental illnesses; 19% could only name one; and 60% could name two or more. The mean number of correct mental illnesses cited was 2.1 (SD 1.8). Schizophrenia was the most commonly known type of mental illness (74%). Other known mental illnesses were depression (39%), manic depression (19%), and paranoia (13%) [19].

In Sri Lanka a study was designed to categorize aspects of mental community health literacy in terms of ability to recognize problems, helpful interventions, helpful referral options, and outcomes in a target adolescent population [20].

In another study conducted in Agaro, Ethiopia, aimed to assess how mental health problems were perceived by a community. Schizophrenia was identified (74%) by a significant number of people as a mental health problem. In this study, 58%, 29%, and 15% of the respondents identified epilepsy, generalized anxiety disorder, and major depressive disorder (MDD), respectively, as mental health problems. A total of 728 people were interviewed with a response rate of 99.5% [21].

To determine the levels of mental health knowledge, different methods are being used. The Mental Health Knowledge Schedule (MAKS) score is a mental health knowledge scales that is widely used in developed countries to measure or determine level of mental health knowledge. One study conducted at the University of Tennessee used the MAKS tool (Canadian Psychiatric Association, Ottawa, ON, Canada) to find the overall knowledge of mental health; the highest MAKS score was 30, and the mean score among participants was 25.88 (2.25) [22]. A relatively similar finding was observed in a study conducted in Ireland. Results of this study indicated that attitudes among the study sample were generally positive [23].

The factors associated with a statistically significant higher total knowledge score on the MAKS were identified in another study conducted in England. According to this study, factors such as female gender, higher socioeconomic status, and knowing someone with a mental health problem were found to be strongly associated with higher total knowledge score on the MAKS [24].

### 2.3. Knowledge and Beliefs about Appropriate Help Seeking

MD is associated with stigma, and this may affect seeking help. For instance, in the Germany there is a greater reluctance in discussing MD with relatives and friends as compared to discussing physical disorders [25]. It was found by the national Italian survey that 99% of the study participants thought that the best way to recover from depression was to find help from the outside [26].

There are other conducted studies in other European countries which gained similar findings. Based on a study in the United Kingdom, most respondents (70%) would contact a general practitioner (GP) if a friend or neighbor was showing signs of mental illness. Other important contacts were: social workers (26%), hospitals (24%), police (22%), the patient’s family (22%), and one of the patient's friends (14%) [27].

Unlike the pro-psychiatric treatment findings from developed countries, one study conducted in Delhi, India, highlighted adverse attitudes towards seeking psychiatric treatment for mental illness [28].

In another study conducted in Agaro town, Ethiopia, respondents (76%, 83%, 72.4%, and 72.5%) preferred modern medicine for the treatment of epilepsy, schizophrenia, major depression, and generalized anxiety disorders, respectively. About 21% and 19% of the respondents preferred holy water for the treatment of epilepsy and schizophrenia, respectively [6].

### 2.4. Community Reaction to People with Mental Illness (MI)

There is higher risk in mental illness often for those affected because of stigmatization by members of the community [29]. It has been concluded in studies in North America and Western Europe that stigma is a major problem in the community [30,31,32].

In Iraq, one study aimed to assess public perception of mental health. Their results revealed that around half of respondents believed people with mental illness should not get married and have children. While just under half believed people with mental illness should be limited and have no contact with others [33]. There are similar results reported from Tehran, Iraq which all respondents (*n* = 123) reported that they had experienced feelings of alienation, discrimination, and social withdrawal [34].

Other studies in Africa also suggested that stigma is a common experience faced by people with mental illness. Results from the study in Zambia revealed that stigma towards people with mental illness is harmful across Zambian society [35].

The Agaro, Ethiopia study showed that there was a positive attitude concerning the four mental health problems by respondents towards work opportunities, marital prospects, chance for education and cure by modern medicine. Epilepsy was considered as the most serious problem, followed by schizophrenia. The least serious problem was the major depressive disorder [6].

### 2.5. Community Prevalence and Associated Factors of Common Mental Disorders

A high prevalence of psychiatric disorders worldwide was consistently reported by epidemiological studies, especially in developing countries [2]. Effective treatment is not provided for disorders including insomnia, anxiety, fatigue, irritability, depressive moods, difficulty concentrating, and somatic complaints, but they very common issues among adults and are quite often overlooked [2,36,37,38].

The group of common mental disorders (CMD) has been compromised by the set of symptoms [2]. CMDs refer to conditions of psychic distress that meets the nosological criteria of the International Statistical Classification of Diseases and Related Health Problems (ICD-10). The Diagnostic Statistical Manual (DSM) for the most prevalent disorders includes the groups of anxiety, depressive, and substance abuse disorders (mainly alcohol), in their mild or even moderate forms. These are well-known causes of major functional incapacity, and are often as significant as those seen in well-established psychiatric conditions [38].

In Santiago, Chile a cross-sectional survey of private households aimed to investigate the prevalence of common MD and socio-demographic correlates among adults. Their results revealed that the most common symptom was ‘worries’, with a prevalence of 43%; other non-specific symptoms were also common (e.g., fatigue, irritability and sleep problems) [39].

To assess common mental health problems, another study was conducted in Agincourt, the rural Limpopo Province of South Africa, and in Khayelitsha, a Peri-urban township near Cape Town. Their main approach was to use self-report instruments (SRQ) in two random population samples and among respondents at primary care and traditional healer settings [40].

Another study in a rural district of Kenya also showed that the point prevalence of common mental disorders (CMDs) in the study sample of 1000 households was 10.8%, largely comprising mixed anxiety depression (6.1%), panic disorder (2.6%), generalized anxiety disorder (1.6%), and depressive episodes (0.7%) [41].

Likewise, two studies from Ethiopia assessed the prevalence of common mental distress and its factors among residents of Jimma and Kombolcha towns. To determine the prevalence of CMD, both studies followed a community based cross sectional study design and used a Self-Reporting Questionnaire (SRQ). The results of both studies indicated that there is a high prevalence of CMD among different groups of the society [42].

Overall, as we can see from the aforementioned empirical evidences there are numerous studies on community prevalence to MD and community health knowledge, perceived beliefs and associated factors of MD and mental illness in different parts of the world both in developing and developed countries setting. However, to best of our knowledge there is a lack of systematic studies on this topic in Ethiopia and they are almost nonexistent in the Tigrai region context. Considering this gap hence, the study aims to address the following research questions:

1. What is the level of community health prevalence of MD?
2. What are the associated risk factors of MD among the community in Mekelle city?
3. What is the level of community mental health knowledge?
4. What are the key factors associated with good mental health knowledge?
5. How are the causes/risk factors and preferred sources of help for MD perceived among community in Mekelle city?

## 3. Materials and Methods

### 3.1. Background of the Study Area

This study was conducted in Mekelle City (the capital of Tigray region), Ethiopia. Mekelle is located in the northern part of the country, 783 km from Addis Ababa (the capital city of Ethiopia). The total population of the city was 215,546 people as reported by 2009 Central Statistics Authority (CSA) data. Females constitute 51.4% of Mekelle’s population and males represent 48.6%. The population under the age of 25 constitutes 63% of the total population. Twelve percent of the population are children under the age of 5. Children of school age constitute about 38% of the population; in Mekelle these children are aged between 4 and 18. Fifty-two percent of the female population are women of reproductive age. Sixty percent of the total population is in the labor force. Five percent of the population are elderly. It is expected that by 2015, assuming a medium growth rate of 2.7%, the population of the city will be 332,013 people [43].

**Figure 1:**
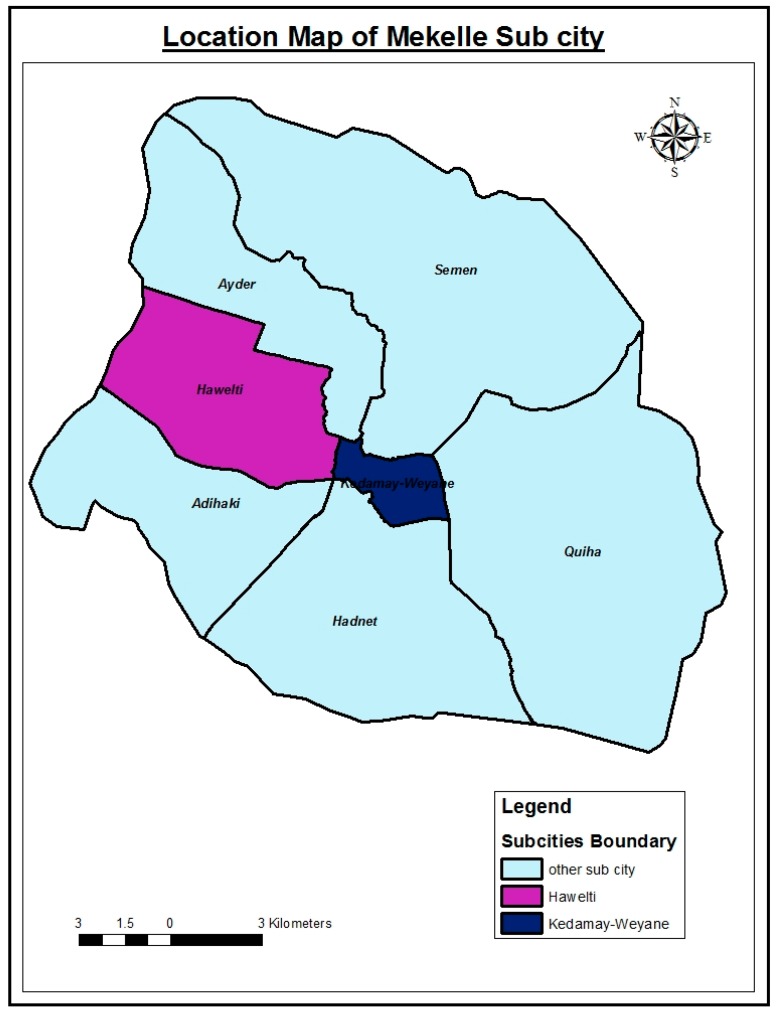
Map of Mekelle city showing sub locations or sub-cities.

Overall, there are 7 sub cities (SC), 35 tabias and 105 ketenas in Mekelle city. The main sub-sub cities comprise Ayder, Kedamay Weyane, Hadnet, Quiha, Adi-haqi, Hawelti, and Semien (see Figure 1). In this study two sub-cities were selected, namely Kedamay Weyane and Hawelti. Based on the information obtained from the health offices of these two sub-cities, the estimated population and household data is as presented in Table 1.

### 3.2. Survey Design and Sampling

A community-based cross-sectional study was conducted in the purposely selected two sub-cities of Mekelle city, namely Kedamay-Woyane and Hawelti. This is done because according to the Mekelle city’s health desk office there is a relatively higher level of poverty in the aforementioned sub cities compared to the other sub cities. It is known in the literature that MD is associated with the level of poverty.

**Figure 2:**
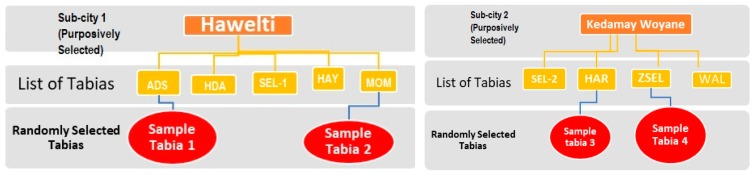
Schematic presentation of the sampling procedure of the study.

In selecting the sample respondents, then, four tabias (two tabias from each sub cities) were randomly selected using simple random sampling method. Accordingly, a sample household is selected using a systematic random sampling technique with a sampling interval of 6 and the household heads were automatically selected as a study sample. According to probabilities proportional to the size technique the sample size was distributed by each tabias. The list of households obtained from the sub-city administration in the selected tabias was used as a sampling frame. Figure 2 presents the sampling procedure used in the study.

### 3.3. Sample Size

According to studies conducted in other parts of the Ethiopia, the prevalence of CMDs was found to be 11.7% in Addis Ababa and 19.3% in Haromaya [44,45]. In this study the sample size was calculated using the prevalence rate of 11.7% to obtain a maximum sample size at 95% certainty, ±4% error margin (because the estimated proportion is <20%) and a 5% non-response rate. This provided a sample size of 260. See the steps for sample size computation below.

(1) $$\;n\;=\;\frac{1.96^{2}\;p\left(1-P\right)}{d^{2}}\;$$

(2) $$\;n\;=\;\frac{{\left(1.96\right)}^{2}\;0.117\left(1-0.117\right)}{{\left(0.04\right)}^{2}}\;$$

*n* = 248.04. The calculated sample size is 248.04 ≈ 248, and non-response rate (5%) is 12.4 ≈ 12. The final required sample size for this study became 260 (248 + 12 = 260).

where: 

*n* = sample size

P = Life time prevalence of MD = 11.7%

Q = 1 − P

*d* = Margin of error between the sample and the population = 0.04

$Z_{2}^{a}$ = Critical value at 95% confidence level of certainty (1.96)

### 3.4. Data, Data Collection Tools, and Procedures

A quantitative data has been collected from a randomly selected 260 households constituting 83 households from Kedamay Woyane sub city and 177 households from Hawelti sub city. In this study the source population included adults aged 18 and above who lived in Mekelle city for at least 6 months or more. Individuals who were found to be too seriously ill or cognitively disabled to give consent, and those who were unable to hear and recall events were excluded from the study.

Generally a structured questionnaire was used to collect the primary data. The questionnaire was initially prepared in English and later translated in to Tigrinya. The content of the questionnaire included socio-demographic variables, MAKS to measure mental health related knowledge, variables designed to assess the perceived causes of MD and the preferred sources of help for MD, questions related to community reaction towards people with mental illness and SRQ-20 items to determine the community prevalence and associated factors of MD.

In the current study to assess the prevalence and presence of MD, the Self-reported questionnaire (SRQ-20) items were adopted (developed by WHO)*.* Originally, the SRQ was designed as self-administered scale. Because of the low literacy rate in developing countries it is also suitable for interviewer-administered questionnaires. Each of the 20 items is scored 0 or 1. The presence of symptom during the past month is showed by score of 1. The absence of the symptom is showed by score of 0. In developing countries the most commonly used cut-off point is 7/8 (7 “yes” responses represent a non-case, while 8 “yes” responses represent a case. It was used in previous community-based studies in Ethiopia [46]. The validity study of SRQ-20 by Youngman showed that specificity (83%) and sensitivity (89.5%) were optimum at 7/8 cut-off points. In this study a cutoff point of 7 and above was taken as having a MD based on findings in other parts of the country [19]. In the last step, the internal consistency of the SRQ-20 item in this study was measured and it provided a reliability coefficient of 0.698 which indicates that the items have acceptable level of internal consistency.

The mental health knowledge schedule (MAKS) was another standardized tool adopted and used for this study. 12 items are comprised by MAKS. There are six items of stigma linked with mental health literacy areas, including help seeking, ability to give advice, support, employment, treatment, and recovery. And also six items assessing knowledge of mental illness diagnoses. A 5-point scale was used to show total disagreement (scale of 1) and strong agreement (scale of 5). Better mental health literacy was indicated by higher scores [47]. Then, a median point of the first 6 items (22.00) was taken to determine the level of mental health knowledge among study participants. The internal consistency for the MAKS items is found to be .449 and this low result might be associated with the second part of the MAKS items were dichotomized.

The finalized and translated questionnaire was converted in to a mobile app (form) to facilitate the data collection using a mobile phone. Of 26 individuals the questionnaire was pre-tested from part of the city which was not included in the sample. Three data collectors (two graduate nurses and one midwife) were employed and trained for 2 days on the questionnaire and the mobile app designed for the data collection. An interview was conducted for the respondents in private setting with the close supervision of the principal investigator. The data extracted from the server was reviewed and checked for completeness before exported to SPSS (SPSS Inc., Chicago, IL, USA) for analysis.

### 3.5. Variables

Both dependent and independent variables were identified and measured to address the research questions. The dependent variables are mental distress and mental health knowledge. Likewise, socio-economic and demographic variables comprising age, sex, occupational status, marital status, religion, educational status, gross family income and family size were taken as independent variables. In addition, behavioral variables such as alcohol use, cigarette smoking, khat use, level of social support, financial difficulty, and previous mental and medical illness history-related variables such as history of mental illness, and history of chronic physical illness were also taken as independent variables. For the measurement attributes of all the variables under consideration see Table 2.

### 3.6. Methods of Data Processing and Analysis

The data that was sent to the server by the data collectors was extracted, checked and exported to SPSS version 20 (SPSS Inc., Chicago, IL, USA) for analysis. Results obtained from questions designed to assess community knowledge and perceived beliefs of MD were interpreted and analyzed descriptively using proportions, percentages, means, range and measures of central tendency. Bar charts and graphs were also used for the interpretation of results. The prevalence of MD was estimated from a cut-off point defined according to the number of positive answers for symptoms. Subjects are classified as suspected of MD cases when they had seven or more “yes” responses for the SRQ-20 items.

Both bivariate and multivariate analysis was conducted in order to explore associations and identify independently linked variables with pattern of MD and mental health knowledge. To do so, each independent variable was separately entered into bivariate analysis. Then, variables with statistical significant association (with *p*-value of less than 0.25) on bivariate analysis were entered into multivariate logistic regression once. Then, it was concluded that variables with *p*-value (less than 0.05 on multiple logistic regressions) had a significant association with MD or mental health knowledge pattern. The strength of association of the variables was determined using odds ratio and 95% confidence level.

### 3.7. Ethical Consideration

Ethical clearance was obtained from Tigrai Regional Health Bureau ethical clearance committee. Written consent was also obtained from all of the respondents participated after the data collectors briefed them all the terms and conditions of participating in this study. To keep the respondents anonymous, respondent unique code was used and it efforts were made to maintain the privacy and confidentiality of the information provided by the study participants. Study participants who were identified as having a MD were advised by the data collectors to contact a psychiatrist or a mental health professional.

### 3.8. Model Specification

In this study to examine community knowledge, perceived beliefs and associated factors of MD, a community-based cross sectional study was conducted in two sub-cities of Mekelle city, Tigrai, Ethiopia. A logistic regression was used to investigate the independent factors associated with MD (outcome variable 1) and the independent predictors of good mental health knowledge (outcome variable 2). Logistic regression is the most commonly used model to examine the effects of the independent predictor variables on binomial outcomes [48]. To predict the independent factors or predictors (X_1_, X_2_, and X_3_) associated with the two binomial dependent variables Y_1_ and Y_2_ that takes values of “with MD” and “without MD” and “poor MHK” and “good MHK”, the following regression function is used as logit (p):

(3) $$\;==log⎣\frac{p\left(y=1\right)}{1-\left(p=1\right)}⎦=\beta_{0}+\beta_{1}x_{1}+\beta_{2}x_{2}+...+\beta_{K}x_{K}\;$$

In the first step of the model building; a univariate analysis was applied to explore the unadjusted association between the independent variables and outcome variables (Y_MD_ and Y_MHK_, respectively) separately. To do so, each of the 16 independent variables was included in a logistic regression model, one for each time. The univariate model is therefore constructed as follows:

(4) $$\;log\;odds[y=\frac{1}{x_{1}}]=log⎣\frac{p\left(y=\frac{1}{x_{1}}\right)}{1-p\;\left(y=\frac{1}{x_{1}}\right)}⎦=\beta_{0}+\beta_{1}x_{1}\;$$

Based on the results of the univariate analysis, variables with *p*-value of smaller than 0.25 were included for further multivariate analysis. The cutoff value of 0.25 is recommended by different studies [49].

In the second step, variables identified in the first step were fits in to the multivariable model. Then variables that failed to contribute to the model are eliminated and the two models are compared using partial likelihood ratio test.

Finally, goodness of fit test was conducted to check the fit of the model. Then the Hosmer-Lemeshow (H-L) inferential goodness-of-fit test was applied for this purpose.

## 4. Results and Discussion

### 4.1. Community Prevalence of Mental Distress

**Figure 3:**
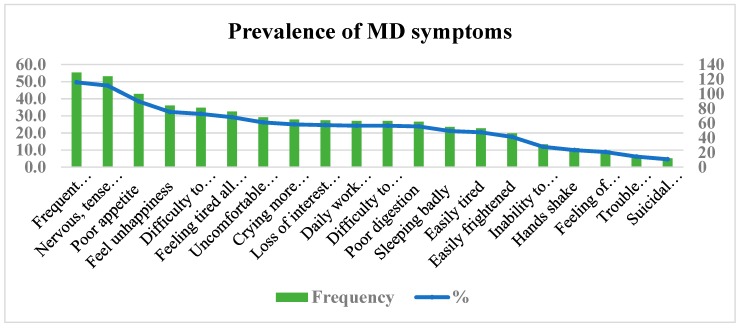
Prevalence of mental distress (MD) symptoms among study participants in Mekelle city, Tigrai, Ethiopia.

The point prevalence of MD in this sample was 25.4% (95% CI: 20.4–30.9) determined using the SRQ-20 items with a cutoff point of 7 and above. The most prevalent symptoms were frequent headaches (129:49.6%), feelings of nervousness, tenseness, and wariness (124:47.7%), and poor appetite (100:38.5%) The least reported symptoms of MD were suicidal intentions (12:4.6%), trouble thinking clearly (16:6.2%) and feelings of worthlessness (23:8.8%) (see Figure 3).

### 4.2. Factors Associated with Mental Distress

After adjusting the confusing factors on multivariate logistic regression analysis, findings from Table 3 showed that the likelihood of MD was about two folds higher among females as compared with males (Adjusted Odds Ratio AOR = 1.538, 95% CI: 0.648–3.651). Occupational status of study participants was also found to have a significant statistical association with MD. Employed and self-employed participants had a six and five times (AOR = 6.076, 95% CI: 1.461–25.280 and AOR = 4.785, 95% CI: 1.214–18.859) increased chance of having a MD, respectively, as compared to housewives.

Daily alcohol use was significantly associated with the risk of developing a MD. Study participants who reported daily consumption of alcohol had a five times increased risk of developing MD compared to non-users or who reported never use of alcohol (AOR = 5.131, 95% CI: 1.614–16.314). Regular use of khat was also found to be a risk factor for MD. It is found that regular or daily khat users had a five times higher chance of experiencing MD than non-users of khat (AOR = 8.122. CI: 1.232–53.551). History of accident or traumatic life events was significantly associated with MD. Accordingly, study participants who reported experience of accident or traumatic life events during the last one year found to have a three times increased risk of developing MD as compared to study participants who report no traumatic event or experience in the last one year (AOR = 3.313, 95% CI: 1.557–7.048). Similarly, previous mental illness diagnosis and treatment found to be significantly associated with MD. Study participants with previous history of mental illness were having 11 times (AOR = 11.239, 95% CI: 2.065–61.17) higher MD than study participants with no history of mental illness diagnosis or treatment.

Variables such as age, level of education, marital status, religion, gross family income, family size cigarette smoking, and feelings of financial insecurity and level of social support showed no significant statistical association with MD.

### 4.3. Mental Health Knowledge of Study Participants

Table 4 shows study participants’ responses to Mental Health Knowledge Schedule items (MAKS part A and B). According to the findings from the first part of MAKS items, 122 (46.9%) of the study participants do not approve of notion that most people with mental health problems should have paid employment. One hundred and twenty four participants (47.6%) agree that people with mental illness can have a paid job or employment. A large majority of the study participants (219; 84.2%) gave a positive response to the item “I know what advice to give a friend to get professional help, If she (he) has a mental health problem,”. Similarly, the idea that medication could be an effective treatment for people with mental health problems was favored by the majority of the study participants (213; 81.9%) while 41 (15.7%) were not in favor the idea. In addition, 224 (86.1%) of the participants were agreed with the idea that psychotherapy (e.g., talking therapy or counseling) could be an effective treatment for people with mental health problems, while the remaining 20 (8.8%) expressed their disagreement. Around 224 (86.1%) of the study participants strongly expressed that people with severe mental health problems could fully recover, while 48 (18.4%) of them show their strong disagreement with the idea. The idea that most people with mental health problems went to a community healthcare professional to get help was favored by 153 (58.8%) of the study participants, while 88 (33.8%) of the participants responded that they did not think or agree that most people with mental health problems should go to a healthcare professional to get help.

In the second part of MAKS, the participants were asked to classify various conditions as mental illness. To this effect, 147 (56.5%) of the study participants ¡failed to recognize depression as a mental illness, while the remaining 113 (43.5%) of the study participants recognized depression as a mental illness. Stress was classified by the majority of the study participants (216; 83.1%) as a mental illness and only 44 (16.9%) of the respondents classify stress as a non-mental illness condition. In total, 234 (90%) of the study participants responded that schizophrenia is a mental illness while the remaining 26 (10%) of the respondents recognized schizophrenia as a non-mental illness condition. Bipolar disorder (manic-depression) was recognized by 226 (86.9%) study participants as a mental illness, while the remaining 34 (13.1%) of the participants responded that it is not a mental illness or a mental health condition. In addition, 169 (65%) of the participants were able to recognize drug addiction as a mental health condition, but a significant number of study participants (91; 35%) failed to recognize drug addiction as a mental health condition. Grief was classified as a mental health condition by 59 (22.7%) of study participants. However, a majority of the study participants (201; 77.3%) were able to recognize grief as a non-mental health condition.

### 4.4. Factors Associated with Mental Health Knowledge (MHK)

Table 5 shows the final model adjusted for potential confounders to recognize independent predictors associated with MHK. In this model, male participants were nearly two times more likely to have a better mental health knowledge than female study participants (AOR = 1.836; CI = 1.059–3.182). A higher level of education was found to be strongly associated with good mental health knowledge. Study participants with diploma and above level of academic qualification had a four times higher level of mental health knowledge than that of illiterate study participants (AOR = 4.180; CI = 1.429–12.224). Study participants who reported having a strong level of social support found to have nearly 3 times increased chance of having a better mental health knowledge than those who reported that they had poor level of social support (AOR = 2.819; CI = 1.248–6.368).

### 4.5. Perceived Beliefs about Causes of MD & Preferred Source of Help for MD

#### 4.5.1. Perceived Causes of Mental Distress

**Figure 4:**
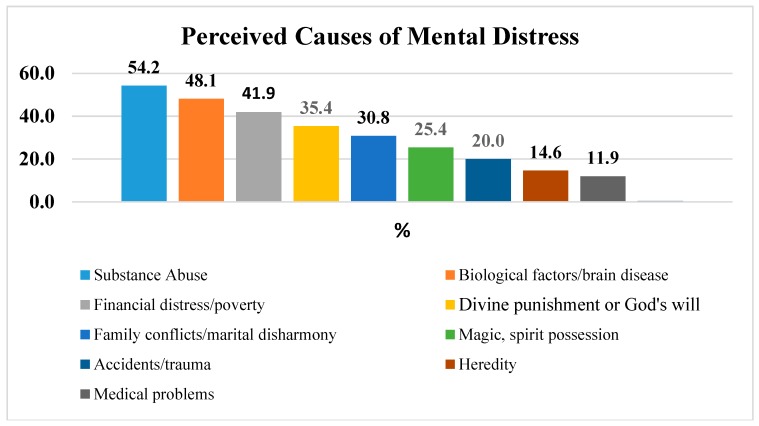
Most commonly reported causes of mental distress (*n* = 260).

More than half of the study participants (141; 54.2%) expressed the view that substance abuse or misuse could cause MD (see Figure 4). The second most commonly endorsed cause of MD was found to be biological factors or brain disease. In total, 125 (48.1%) of the study participants responded that MD can be caused by biological reasons or factors that are related with brain diseases. The third most commonly cause of MD reported by study participants was financial distress or poverty. Divine punishment or God’s will was reported by 92:35.4% of the study participant as a cause of MD. Heredity (38:14.6%) and medical problems (31:11.9%) were the least endorsed causes of MD.

#### 4.5.2. Preferred Sources of Help for Mental Distress

**Figure 5:**
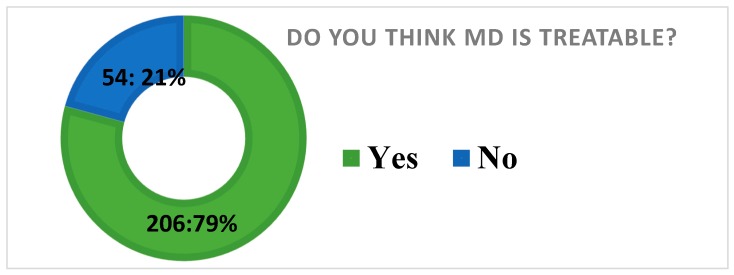
Participants perception about the treatability of mental distress.

It was required that the study participants respond to the following question “do you think mental and behavioral disorders are treatable?” to assess their perception before they are asked to report their preferred sources of help for MD. Accordingly, the majority of the study participants (206; 79%) reported that mental and behavioral disorders can be treated or people with MD can fully recover or be cured. However, 54:21% of the participants had a perception that mental and behavioral disorders are not treatable or people with MD or illness cannot fully recover or cured (see Figure 5).

**Figure 6:**
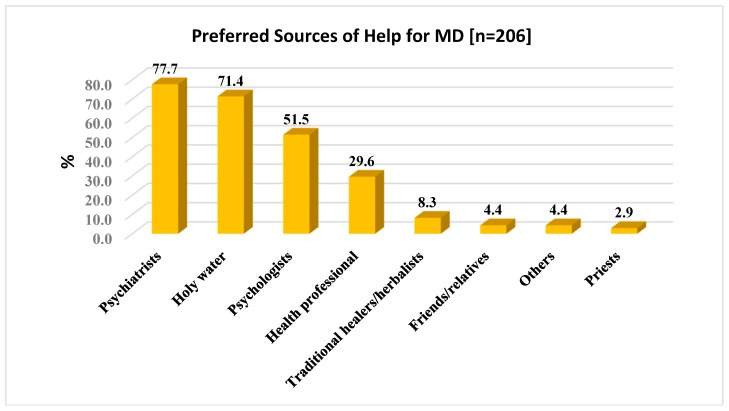
Preferred sources of help for MD (*n* = 206).

It was required that the study participants provide a report about their preferred sources of help for MD (206; 79%). As indicated in Figure 6, psychiatrists were the most preferred sources of help for MD followed by holy water and psychologists. They were reported by 160 (77.7%), 147 (71.4%), and 106 (51.5%) of the study participants, respectively, as preferred sources of help for MD. Community health professionals also preferred by 70 (29.6%) of the study participants as preferred sources of help for MD. Friends or relatives and priests were the least preferred sources of help for MD problem. 9 (4.4%) and 6 (2.9%) of the study participants preferred friends or relatives and priests as a source of help for MD, respectively.

## 5. Discussions

### 5.1. Community Prevalence and Associated Factors of Mental Distress

The community prevalence of MD in this community based study was found to be 25.4% (95% CI: 20.4–30.9). A similar community based study conducted in Kombolcha, Ethiopia, using a similar screening tool and cutoff point found a prevalence of 32.4% with 95% a confidence interval of (30.3–34.5%) [50]. Another study conducted in Jimma town, Ethiopia, found a community prevalence of 33.6% [42]. The prevalence of the current study was found to be lower than the studies conducted in Kombolcha and Jimma towns. The likely reason could be the sample size for this study was lower than those studies (a total of 526 (Kombolcha) and 745 (Jimma) individuals were participated). A study conducted in the Yoruba speaking parts of Nigeria showed a lower prevalence of MD (12.1%) compared to the present study but unlike the present study, the Nigerian study used the World Mental Health version of the Composite International Diagnostic Interview (WMH-CIDI) to determine the prevalence of MD [9].

The current study showed that the likelihood of MD was nearly two-folds higher among females as compared with males regarding the factors associated with MD, (AOR = 1.538, 95% CI: 0.648–3.651). Other studies conducted in different parts of the world and Ethiopia such as South Africa [40], south west and northern parts of Ethiopia [42,50] and Chile [39] obtained a similar result.

This study revealed that factors such as being unemployed, self-employment and employment at the government or other organization were found to have a significant association with MD. This finding is somehow in agreement with findings of the study conducted in South Africa, Khayelitsha, that showed being unemployed (AOR 2.70; 95% CI 1.25–5.84) is strongly associated with MD [40]. Contrary to the findings of the current study, a study conducted in Addis Ababa found that employment was inversely associated with risk of MD [44]. Job stress can be attributed to the increased risk of MD among employed and self-employed study participants of this study. Overwork, lack of clear instructions, unrealistic deadlines, lack of decision-making, job insecurity, isolated working conditions, surveillance, and inadequate child-care arrangements are considered as some potential causes of work related stress [51].

The current study revealed that daily alcohol use was significantly associated with the risk of developing a MD. Study participants who reported daily consumption of alcohol had five times increased risk of developing a MD compared to non-users or participants who reported never use of alcohol (AOR = 5.131, 95% CI: 1.614–16.314). Similarly, a study conducted in Kombolcha town, Ethiopia, found that current alcohol drinkers had an increased chance of developing common mental distress or MD than non-alcohol drinkers [50]. Alcohol counselling program combined with social and family support plays an essential role in alcohol control and prevention of psychiatric comorbidity [52]. Regular khat use was also found to be a risk factor for MD in the current study (both studies conducted in Jimma and Kombolcha supported this finding) [42,50]. According to the findings of the study conducted in Jimma town, khat use was strongly associated with MD (AOR = 1.56, 95% CI: 1.14–2.13) [42].

Based on the current study findings the participants who reported experience of accident or traumatic life events during the last one year found to have a 3 times increased risk of developing MD as compared to study participants who report no traumatic event or experience in the last one year (AOR = 3.313, 95% CI: 1.557–7.048). Study participants with previous history of mental illness were also having 11 times (AOR = 11.239, 95% CI: 2.065–61.17) higher MD than study participants with no history of mental illness diagnosis or treatment. However, one study done by Yim (2015) [53] in Hong Kong indicated that there was no statistically significant difference on MD prevalence between the groups with and without a previous psychiatric assessment.

Strong level of social support was found to be inversely associated with the risk of MD. This finding is in line with the findings of the Kombolcha study [50].

### 5.2. Mental Health Knowledge and Predictors of Good MHK

To measure the mental health knowledge of the study participants the current study employed a mental health knowledge schedule (MAKS) items. According to this study the mean total score for the MAKS Part A (6 items) was 21.4308 (SD = 0.13997, 95% CI: 21.0527–21.7612). Results indicated that 154:59.2% of the study participants had a poor knowledge of mental health (median score <22), while the remaining 106:40.8% of the study participants demonstrated that they have a good knowledge of mental health (median score >22) Since there are no similar studies using MAKS method in Ethiopia and other developing countries, it was mandatory for the current study to crosscheck the findings with studies conducted in developed country. To this effect, a study conducted at Walden University to evaluate the effectiveness of an educational mental health program found a mean total mask score of 24.46 (SD = 2.42) which is relatively higher than the results obtained by this study [54]. The disparity might be explained by different mental health education programs implemented to improve community mental health knowledge in developed countries. The main core of this study was to assess the effectiveness of mental health education program. Similarly, another study conducted at the University of Tennessee showed that the highest MAKS score of 30 and the mean score among participants was 25.88 (2.25). The median and mode was 26. The minimum score was 20 and the maximum was 30 [22].

The current study reported a relatively higher level knowledge as compared to a study conducted in Dodoma Municipality, Tanzania. The results of this study showed that most of the study participants 85.9% (*n* = 330) had poor knowledge about mental illness [55]. This could be due to the disparity in the academic qualification of the study participants of these two studies.

The second part of the MAKS was designed to assess study participants ability to recognize a mental disorder. The current study revealed that 147 (56.5%) of study participants failed to recognize depression as a mental illness while the remaining 113 (43.5%) of the study participants recognize depression as a mental illness. Similarly, due to the limited application of the MAKS score, the findings of this study was compared with the results of other studies obtained from using a different approaches. In a study conducted in Gimbi, Ethiopia, only 29% of the respondents identified Major Depressive Disorder as a mental health condition [21]. The huge disparity might be due to using a vignette of the condition based on the ICD-10 criteria while this study asked the participants to say “true” or “false” by just naming the condition without providing detail description of the condition. Another comparative study conducted in Australia and Japan revealed that 65.3% of the Australia and 22.6% of the Japan participants were able to recognize the depression vignette correctly [19].

Similarly stress was categorized by the majority of the study participants (216:83.1%) as a mental illness and only 44 (16.9%) of the respondents classified stress as a non-mental illness condition. This could be due to the fact that the equivalent Tigrinya term for stress *(ጭንቀት)* might be confusing for the study participants. In total, 234 (90%) of the study participants responded that schizophrenia is a mental illness while the remaining 26:10% of the respondents recognized schizophrenia as a non-mental illness condition. However, in the study conducted in Agaro, it was found that 74% of the participants identified schizophrenia as a mental health problem [21].

It was found in the current study that 169:65% of the participants were able to recognize drug addiction as a mental health condition. However, a significant number of study participants (91; 35%) failed to recognize drug addiction as a mental health condition. Study participants with diploma and above level of academic qualification were fourtimes higher level of mental health knowledge than that of illiterate study participants. The mentioned findings lacks adequate support from other study findings but educational level was found to be associated with better recognition of mental illness in a study conducted in Sri Lanka [20].

### 5.3. Perceived Beliefs about Causes of MD & Preferred Source of Help for MD

#### 5.3.1. Perceived Causes of MD

This study revealed that more than half of the study participants (141:54.2%) expressed the view that substance abuse or misuse could cause MD. The second most commonly endorsed cause of MD was found to be biological factor or brain disease (125:48.1%) and financial distress or poverty was reported as the third most commonly cause of MD. Divine punishment or God’s will was reported by 92:35.4% of the study participant as a cause of MD. This finding was somehow in line with the findings of study conducted in Karifi Vilage, Nigeria. The study reported misuse of drugs as the most commonly endorsed cause of MD followed by divine punishment/God’s will and magic or sprit possession [17].

In addition, a study conducted in Teheran found a different result compared to the current study. The Teheran study indicated that stress, traumatic physical events and psychological trauma were among the most frequently reported causes of MD and the will of God and evil spirits were the least reported causes of MD [34]. The discrepancy between the findings of present study and the Tehran one might be attributed to the difference at the level of mental health knowledge between the two study populations.

#### 5.3.2. Preferred Sources of Help for MD

To examine the preferred sources help for MD, the study assessed the perception of the study participant about whether MD is treatable or not and it has been found that majority of the study participants (206; 79%) reported that mental and behavioral distress can be treated or people with MD can fully recover or be cured. However, 54:21% of the participants had a perception that mental and behavioral disorders are not treatable. A study conducted in Delhi, India reported that 40% of the urban samples or higher than the current sample believed that mental illness or MD is untreatable [17].

Concerning the preferred source of help for MD, psychiatrists were the most preferred sources of help for MD followed by holy water and psychologists. They were reported by 160: (77.7%), 147 (71.4%), and 106 (51.5%) of the study participants respectively as preferred sources of help for MD. This finding is supported by studies conducted in developed countries such as Italy. The Italy study reported that psychologists (55%), primary care physicians (PCPs, 38%), psychiatrists (29%), and neurologists (21%) as the most preferred sources of help [26]. In a similar study conducted in Agaro, Ethiopia, Holy water was preferred by 21% of the study participants [6], which is way lower than the current study result and it might be due to the fact that the study sample for this study is largely dominated by Orthodox Christian followers (82.3%).

According to another study done by Walters et al. (2008) [56], the most popular sources of help endorsed by study subjects were talking to family or friends (59%), advice from GP or nurse (34%), relaxation or yoga (29%), talking therapy (29%), and exercise, sport, or hobbies (28%). However, the current study doesn’t include relaxation or yoga and exercise or sport as preferred sources of help assuming which these activities are less common in the study area context and as a treatment preference for MD. In addition, this study did not explore health-related applications on mobile phones or mobile-health (mHealth) applications which appear to be a feasible approach for disease and health management, especially among young adults [57]. However, advice from GP or nurses, and talking therapy or psychotherapy were preferred by a significant number of study participants in the current study.

## 6. Conclusions

This study indicated that MD is prevalent among community in Mekelle city and frequent headaches, feelings of nervousness, tense and wariness, and poor appetite were found to be the most frequently endorsed or reported symptoms of MD. The likelihood of having a MD was found to be higher among study participants who were female, employed, self-employed, and daily alcohol and khat users. History of accident or traumatic life events, and previous mental illness diagnosis and treatment history are also key risk factors for MD. The protective factor from MD was found to be the strong level of social support.

The study also revealed that the low level of mental community health knowledge among study participants. It was demonstrated by the more than half of the study participants that they had poor knowledge of mental health. Factors such as being a male gender, having a high level of education, having a strong level of social support and having a perception that “MD is treatable” were found to be independent predictors of good mental health knowledge.

According to the evidence, the role of social support to improve mental health or protect from developing a mental illness can be explained in either of the two ways. Firstly social support has a positive effect on mental health regardless of whether or not the individuals are under stress. Secondly they work as a stress buffer; in other words social supports by acting as a buffer or moderator enhance the wellbeing of people under stress.

This study also found that history of accident or traumatic life events and previous mental illness diagnosis and treatment history were significantly associated with MD. However, these two factors were not assessed by the studies reviewed for the purpose of this study. In addition to assess level of mental health knowledge, the current study assessed predictors of mental health knowledge. According to this study, it was found that male participants were nearly two times more likely to have better mental health knowledge than female study participants; higher level of education also found to be strongly associated with good mental health knowledge.

There was a positive perception regarding treatment outcome of mental illness in community. Mental hospital has been identified as a preferred place by the participants for treatment. This indicates preference for specialized care for patients with mental illness.

Therefore, a comprehensive community-based mental health promotion and communication program is needed to ensure the effective implementation of community based mental health care and prevention activities. To this end, enhancing the engagement of community level platforms in terms of program planning, implementation, and evaluation is vital. The stigma attached with mental illness will be decreased by health education and increase in public awareness regarding factual information about mental illness. These measures also improve the help-seeking behavior of the community and lead to reducing burden of psychiatric morbidity.

In addition, since mental health is already integrated in to primary health care package, building the capacity of health extension workers and health care professionals is necessary to improve the quality of mental health prevention, care and treatment services. This study suggests that Information education communication (IEC) programs should be launched to teach the community on the causes, symptoms, treatability of mental health problems and the roles of people with mental health problems in the society. Furthermore, future research works need to investigate this topic using similar assessment instrument (SRQ-20 and MAKS) in a similar community setting and with a relatively larger sample size to find more about the community knowledge, beliefs and associated factors of MD. Although the current study was carried out in Ethiopia, the goal of improving mental health literacy is seen as important in many countries. The current findings suggest that, considering adequate time and investment of resources, it is possible to achieve significant changes in population mental health literacy. Now the main question is how these changes translate into enhancements in population mental health. Therefore, to investigate these changes, there is a need for further monitoring of population mental health.

## Figures and Tables

**Table 1:** Estimated number of population and households in the selected sub cities.

Name of Sub-City	Name of Tabias	Estimated Number of Population	Estimated Number of Households *
Hawelti	Adishumduhun (ADS)	16,337	3713
	Hidasie (HDA)	18,651	4230
	Selam (SEL-1)	7332	1666
	Hayelom (HAY)	12,959	2945
	Momona (MOM)	15,388	3497
**Total**		70,667	16,051
Kedamay Woyane	Selam (SEL-2)	8850	2011
	Haraya (HAR)	6095	1385
	Zesellasie (ZEL)	8581	1950
	Walta (WAL)	9365	2128
**Total**		32,891	7474

Source: Health offices of the respective sub-cities, March 2018. Note: * Household number is estimated using 4.4 as a conversion factor.

**Table 2:** Summary of the descriptive statistics.

Variable Names	Measurement Attributes	Symbols	Valid *n*	Mean	SD	Min	Max
**Outcome Variables**							
Mental distress	1 if received 7 or more “yes”, 0 if received less than 6 or less “Yes” responses for the SRQ-20 items	SRQ_cat	260	0.250	0.436	0	1
Mental health knowledge	1 (good MHK) if received above 22 (median point), 0 (Poor MHK) if received below the median point.	MASK_MEDIAN	260	0.4077	0.49235	0	1
**Independent Variables**							
Sex	1 if male, 2 if female	Gender	260	1.58	0.494	1	2
Age	Continuous variable in number of years	Age	260	36.18	11.420	19	85
Marital status	1 if the respondent is never married, 2 = married, 3 = divorced and 4 = widowed	Maritalstatus	260	1.89	0.758	1	4
Education	1 if the respondent educational status is illiterate, 2 = primary school completed, 3 = secondary or vocational school completed and 4 = college diploma and above.	Educ_cat	260	3.04	1.065	1	4
Religion	1 if the respondent is a follower of orthodox Christian, 2 = Muslim, 3 = Protestant, and 4 = others	Religion_cat	260	1.23	0.565	1	4
Occupational status	1 = unemployed, 2 = employed, 3 = self-employed, and 4 = housewives	Occupation_cat	260	2.82	0.759	1	4
Income	Gross household monthly income in ETB (Ethiopian Birr)	Income	260	4990.3	5380.9	800	50,000
Family size	Number of household members	Familysize	260	3.62	1.781	1	9
Frequency of alcohol use	1 = daily, 2 = 1 or more per week, 3 = occasionally and 4 = non-user	Alchol_use_cat2	260	2.92	1.650	1	4
Cigarette smoking frequency	1 = daily, 2 = occasionally and 3 = non-smokers	Cigarette_cat	260	2.77	0.626	1	3
Khat use	1 = daily use, 2 = 1 or more per week, 3 = occasionally, and 4 = non-users	Khatue_cat	260	3.75	0.705	1	4
History of chronic physical illness	1 if the respondent report history of chronic physical illness and 0 if not.	chronicillness	260	1.79	0.406	0	1
Level of social support	1 = poor, 2 = moderate, and 3 = strong	Socialsupport	260	1.668	0.721	1	3
History of accident/traumatic life experience	1 if the respondent report history of accident or traumatic life events and 0 if not.	Experienceofaccidenttrauma	260	1.79	0.406	0	1
Financial insecurity problem	1 if the respondent report financial insecurity problem and 0 if not.	Financialdifficulty	260	1.52	0.501	0	1
Previous mental illness history	1 if the respondent report history of mental illness diagnosis or treatment and 0 if not.	MentalillnessHistory	260	1.97	0.183	0	1

**Table 3:** Bivariate and multivariate analyses of factors associated with MD (*n* = 260).

Characteristics		^1^ MD		COR (95%–CI)	AOR (95%–CI)
		Without	With		
Sex	Male	73	36	1	1
	Female	121	30	0.503 (0.286–0.884)	1.538 (0.648–3.651) *
Age	16–25	19	6	2.421 (0.533–10.995)	14.494 (1.436–146.244)
	26–34	85	20	1.804 (0.493–6.606)	3.606 (0.499–26.046)
	35–44	50	26	3.987 (1.094–14.528)	3.218 (0.553–18.725)
	45–54	17	11	4.961 (1.196–20.569)	5.572 (0.811–38.291)
	>55	23	3	1	1
Occupational status	Unemployed	4	1	3.917 (0.327–46.899)	12.101 (0.884–165.665)
	Employed	59	29	7.701 (2.209–26.849)	6.076 (1.461–25.280) *
	Self employed	84	33	6.155 (1.791–21.155)	4.785 (1.214–18.859) *
	Housewives	47	3	1	1
Alcohol use	Daily	17	22	6.309 (2.749–14.477)	5.131 (1.614–16.314) **
	1 or more per week	29	8	1.345 (0.520–3.476)	1.963(0.579–6.652)
	Occasionally	70	20	1.393 (0.670–2.897)	1.482 (0.628–3.498)
	Non users	78	16	1	1
Cigarette smoking	Daily	11	17	5.677 (2.497–12.910)	1.879 (0.499–7.070)
	Occasionally	3	0	0.000 (0.000–)	0.000 (0.000–)
	Non-smokers	180	49	1	1
Khat use	Daily users	4	6	5.388 (1.462–19.852)	8.122 (1.232–53.551) *
	1 or more per week	5	5	3.592 (0.999–12.911)	1.771 (.236 -13.309)
	Occasionally	9	6	2.395 (0.813 -7.054)	2.199 (0.509–9.506)
	Non-users	176	49	1	1
History of chronic physical illness	Yes	33	21	2.277 (1.201–4.314)	1.985 (0.926–4.258)
	No	161	45	1	1
Level of social support	Poor	80	42	3.570 (1.300–9.805)	2.615 (0.851–8.035)
	Moderate	80	19	1.615 (0.557–4.679)	1.265 (0.384–4.172)
	Strong	34	5	1	1
History of accident or traumatic life events	Yes	28	26	3.854 (2.041–7.277)	3.313 (1.557–7.048) **
	No	166	40	1	1
Feelings of financial insecurity	Yes	86	40	1.932 (1.093–3.414)	1.697 (0.859–3.349)
	No	108	26	1	1
Previous mental illness diagnosis and treatment history	Yes	3	6	6.367 (1.545–26.233)	11.239 (2.065–61.17) **
	No	191	60	1	1

Notes: ** *p* < 0.01. * *p* < 0.05. ^1^ MD: mental distress.

**Table 4:** Participants responses to mental health knowledge schedule items (* MAKS Part: A and B), (*n* = 260).

**Responses (*n*, %)**					
**MAKS Part A (knowledge)**	**Strongly Disagree**	**Disagree**	**Don’t Know**	**Agree**	**Strongly Agree**
Most people with mental health problems want to have paid employment.	(22) 8.5	(100) 38.5	(14) 5.4	(116) 44.6	(8) 3.1
I know what advice to give a friend to get professional help, if she (he) had a mental health problem.	(3) 1.2	(31) 11.9	(7) 2.7	(176) 67.7	(43) 16.5
Medication can be an effective treatment for people with mental health problems.	(4) 1.5	(37) 14.2	(6) 2.3	(179) 68.8	(34) 13.1
Psychotherapy (e.g., talking therapy or counselling) can be an effective treatment for people with mental health problems.	(0) 0	(20) 8.8	(13) 5.0	(153) 58.8	(71) 27.3
People with severe mental health problems can fully recover.	(8) 3.1	(40) 15.4	(37) 14.2	(149) 57.3	(26) 10.0
Most people with mental health problems go to a healthcare professional to get help.	(8) 3.1	(80) 30.8	(19) 7.3	(150) 57.7	(3) 1.2
**MAKS Part B (recognition of mental disorders)**					
**Conditions**	**True**	**False**			
Depression (True)	(113) 43.5	(147) 56.5			
Stress (False)	(216) 83.1	(44) 16.9			
Schizophrenia (True)	(234) 90	(26) 10			
Bipolar disorder (manic-depression) (True)	(226) 86.9	(34) 13.1			
Drug addiction (True)	(169) 65	(91) 35			
Grief (False)	(59) 22.7	(201) 77.3			

Notes: * Mental health knowledge schedule MAKS 10 © 2009 Health Service and Population Research Department, Institute of Psychiatry, King’s College London.

**Table 5:** Final model adjusted for potential confounders to identify independent predictors to factors associated with MHK (*n* = 260).

Characteristics		* Sig.	EXP (B)	95% of Confidence Interval	
				Lower	Upper
Sex	Male		1		
	Female	0.030	1.836	1.059	3.182
Educational Status	Illiterate		1		
	Primary school completed	0.037	3.419	1.078	10.847
	Secondary school completed	0.131	2.426	0.768	7.655
	College diploma & above	0.009	4.180	1.429	12.224
Level of social support	Poor		1		
	Moderate	0.065	1.731	0.967	3.098
	Strong	0.013	2.819	1.248	6.368
Perception that myocardial infarction (MI) is treatable	Yes	0.005	0.335	0.157	0.715
	No		1		

Note: * Statistical significance: * *p* < 0.05.

## Notes

- *A region* is an ethnic-based administrative territoriality of Ethiopia that is larger than a district.
- *A tabia* is the smallest administrative unit of Ethiopia, similar to a ward or hinterland.
- *A ketena* refers to the smallest urban administrative unit of Ethiopia, similar to a street.
